# Prediction Model for Failed Vacuum Assisted Delivery: A Retrospective Cohort Study

**DOI:** 10.3390/jcm15072522

**Published:** 2026-03-26

**Authors:** Itamar Gilboa, Daniel Gabbai, Lee Reicher, Emmanuel Attali, Yariv Yogev, Anat Lavie

**Affiliations:** Lis Hospital for Women’s Health, Tel Aviv Sourasky Medical Center, Gray Faculty of Medicine, Tel Aviv University, Tel Aviv 6927846, Israel; itamar.glb@gmail.com (I.G.); gabbaidaniel@gmail.com (D.G.); lee.reicher@gmail.com (L.R.); attaliemmanuel@gmail.com (E.A.)

**Keywords:** vacuum assisted delivery, prediction model, cesarean delivery, failure, labor, operative vaginal delivery

## Abstract

**Background/Objectives**: We aimed to determine risk factors and to design a clinically based predictive model for a failed vacuum assisted delivery (VAD). **Methods**: We conducted a retrospective cohort study in a single tertiary university-affiliated medical center between 2011 and 2023. The study population consisted of singleton pregnancies with a VAD trial. The study group comprised cases of a failed VAD, defined as the occurrence of any of the following: (1) more than two vacuum cup detachments; (2) extraction duration exceeding 20 min; or (3) abandonment of the vacuum attempt by the operating physician, with conversion to urgent cesarean delivery (CD). The control group comprised cases of successful VADs. Factors associated with failed VAD were examined by univariate and multivariate analyses. A prediction score was developed to predict failed VAD. A receiver-operating characteristic curve (ROC) was utilized for the model. Internal validation was performed by means of a 70/30 train–test split, with model performance evaluated on the validation set using ROC analysis. **Results**: A total of 131,019 women delivered in our center during the study period. VAD was attempted in 8885 (6.8%) cases, of which 172 (1.9%) failed trials that led to urgent CDs. Several risk factors for a failed VAD were identified, including induction of labor, fetal head station below +2 cm relative to the ischial spines, duration of the second stage of delivery >3.5 h, preeclampsia, birthweight >3750 g, and male gender. The prediction score demonstrated good discriminatory performance, with an AUC of 0.723 (95% CI 0.637–0.810). Internal validation using a 30% holdout cohort revealed that the model maintained good performance, with an AUC of 0.764 (95% CI 0.619–0.909; *p* < 0.001). **Conclusions**: Our model has the potential to assist obstetricians with VAD decision-making and parturient counseling, as well as identifying parturients at high risk for complicated deliveries.

## 1. Introduction

Vacuum assisted delivery (VAD) is used to increase the likelihood of a vaginal birth when maternal or fetal conditions mandate shortening or terminating unassisted labor. The goal of VAD is to avoid the need for a cesarean delivery (CD) and the risks associated with performing a CD when there is full cervical dilatation and fetal head in a low position [1,2,3]. The prevalence of operative vaginal deliveries (OVD) has decreased, particularly in the United States, where rates fell from 9% of all deliveries in 1992 to 3.2% in 2014 [4]. Although VAD is generally thought to be safe, there are some neonatal complications associated with the traction applied to the fetal scalp during the procedure, including lacerations, cephalohematoma, and subgaleal and cerebral hemorrhages. OVD is also anticipated to possibly cause intracranial bleeding as well as neurologic complications [5,6].

While most VADs are successful, the reported failure rate ranges between 2.9–6.5% and may necessitate the use of alternative OVD techniques or an urgent CD [3,7], both of which options have been linked to increased maternal and neonatal risks compared to spontaneous vaginal deliveries [3,8,9]. Alternatively, when VAD is performed successfully, the overall risk is reduced [10], although previous studies that compared maternal and neonatal outcomes of urgent CD versus VAD during the second stage of labor have failed to reach a consensus on the preferred mode of delivery [6,10].

Numerous studies have investigated the risk factors associated with failed VAD [11,12,13], and several have proposed VAD failure prediction models [14,15,16]. Some of them, however, were ineffective in accurately predicting failed OVD [15], while others overlooked various maternal and obstetrical characteristics [14]. As a result, the value of these models in clinical practice is open to question. We aimed to identify the predominant risk factors responsible for a failed VAD and to validate a model based upon clinical parameters for predicting VAD success.

## 2. Materials & Methods

This retrospective cohort study was carried out at a single university-affiliated tertiary medical center between 2011 and 2023. The indications for intervention were a prolonged second stage of labor, a non-reassuring fetal heart rate, or an arrest of descent [17]. VAD was attempted if the fetal head station was engaged and at ischial spines +1 cm. Only singletons with VAD trials were included, and they were divided into a study group of failed VAD deliveries and a control group of successful ones. A failed VAD was defined as the occurrence of any of the following: (1) more than two vacuum cup detachments; (2) extraction duration exceeding 20 min; or (3) abandonment of the vacuum attempt by the operating physician, with conversion to urgent CD. Data from the study and control groups were compared. The exclusion criteria were a previous CD, multiple gestational deliveries, normal vaginal deliveries, and a CD that had not been preceded by a failed VAD.

All medical charts used in the study were derived from computerized delivery room logbooks. Demographic, obstetric, and clinical characteristics included maternal age, parturients without medical insurance, pre-gestational body mass index, height, gestational weight gain, parity, previous CD, mode of conception (spontaneous versus in-vitro-fertilization [IVF]), smoking, pre- and gestational diabetes, gestational age at delivery, onset of labor (spontaneous versus medical induction), epidural anesthesia, premature rupture of membranes (PROM), meconium-stained amniotic fluid, length of the second stage, newborn birthweight, and newborn gender.

The fetal head position during labor (occiput anterior versus posterior) and head station, measured in cm between the leading bony edge and the ischial spines (−2 to +2), were also recorded, with the midpoint (0 station) aligning with the maternal spines [18]. Intraventricular hemorrhage, seizures, skull fractures, subgaleal hematoma, intracranial hemorrhage, neonatal intensive care unit (NICU) admission, and neonatal death were recorded.

Categorical variables were presented as frequency and percentage. Continuous variables were tested for normality by the Kolmogorov–Smirnov test and reported as means ± standard deviation or median and interquartile range. We used the Student *t*-test for normally distributed continuous variables and the Mann–Whitney rank sum test for non-normally distributed continuous variables to compare groups. Chi-square and Fisher’s exact tests compared categorical variables. A multivariable logistic regression model was constructed to identify independent predictors of failed VAD. Variables demonstrating a statistically significant association in the univariate analysis (*p* < 0.05) were included in the multivariable model to account for potential confounding. Subsequently, receiver operating characteristic (ROC) curve analysis was performed to determine optimal cut-off values and to estimate sensitivity and specificity for predicting failed VAD. Model discrimination was assessed using the area under the ROC curve (AUC). Model calibration was assessed using the Hosmer–Lemeshow goodness-of-fit test. A clinical prediction score was constructed by assigning each predictor in the final multivariable model a point value proportional to the magnitude of its regression coefficient (β). Points were derived by rounding each coefficient to the nearest 0.5 and summing the assigned points across predictors to generate an overall risk score. Model performance was evaluated using ROC curves, with calculated AUC and 95% confidence interval (CI). To assess the predictive performance of the model, internal validation was performed using a holdout method. The dataset was randomly divided into a training set (70%) and a validation set (30%). A logistic regression model was constructed using the training data, and predicted probabilities were generated for all participants. Model performance was evaluated on the validation subset by ROC analysis. The optimal cut-off value for the risk score was determined using the Youden index derived from the ROC curve. All statistical analyses were performed using SPSS software (SPSS version 29, IBM, Chicago, IL, USA).

The study was approved by the institutional review board (0284-08-TLV) which waived informed consent due to its retrospective design and anonymized data.

## 3. Results

**Figure 1:**
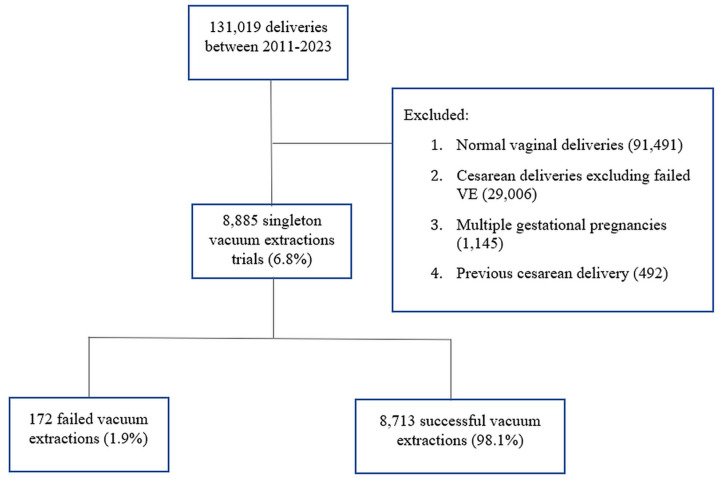
Study cohort flow diagram. A total of 131,019 deliveries occurred during 2011–2023. After excluding cases without a vacuum trial (normal vaginal deliveries, cesareans for other indications, multiple gestations, etc., as detailed), there were 8885 singleton vacuum-assisted delivery (VAD) attempts included. Of these, 172 (1.9%) were failed VADs that required urgent cesarean, and 8713 (98.1%) were successful VADs.

There were 131,019 deliveries during the study period, of which 8885 (6.8%) were VAD. There were 172 (1.9%) failed VAD deliveries (study group) and 8713 (98.1%) successful ones (control group) (Figure 1). The demographic and maternal characteristics are shown in Table 1. The rates of IVF pregnancies, maternal age >35 years, and preeclampsia differed between groups.

The obstetrical characteristics of the study population are shown in Table 2. The univariate analysis revealed that the study group had higher rates of PROM, induction of labor, fetal head station below +2 cm relative to the ischial spines, prolonged second stage of labor > 3.5 h, occiput posterior position, gestational age ≥ 41 weeks, birthweight ≥ 3750 g and a higher proportion of male infants. The use of epidural anesthesia, on the other hand, was more common in the control group.

Neonatal outcomes are presented in Table 3. Given the low event rates for several outcomes, these findings should be interpreted primarily as descriptive. Overall, higher rates of adverse neonatal outcomes, including NICU admission, skull fractures, and subgaleal hematomas, were observed in the failed VAD group.

The multivariable analysis revealed that medical induction of labor (odds ratio [OR] 1.87, 95% CI 1.02–3.43; *p* = 0.044), second-stage duration >3.5 h (OR 2.61, 95% CI 1.28–5.30; *p* = 0.008), fetal head station below +2 cm relative to the ischial spines (OR 2.35, 95% CI 1.33–4.52; *p* = 0.004), birthweight > 3750 g (OR 2.23, 95% CI 1.06–4.70; *p* = 0.035), preeclampsia (OR 7.52, 95% CI 2.77–20.42; *p* < 0.001), and male newborn gender (OR 1.96, 95% CI 1.02–3.78; *p* = 0.043) were independently associated with failed VAD (Table 4).

A scoring system was developed based upon the multivariable logistic regression model to assess the risk of failed VAD (Table 4). Predictors were weighted according to their regression coefficients: a risk score = [0.5 × induction of labor] + [1 × second-stage duration >3.5 h] + [1 × fetal head station below +2 cm relative to the ischial spines] + [1 × birthweight > 3750 g] + [2 × preeclampsia] + [0.5 × male newborn gender].

**Figure 2:**
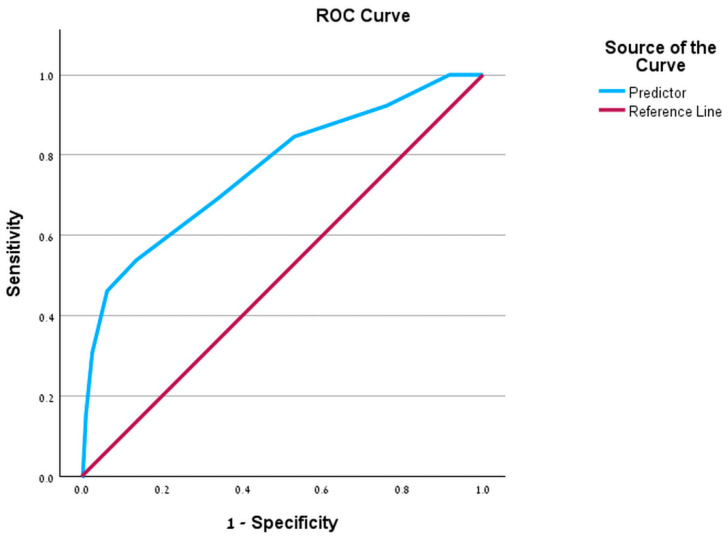
Receiver Operating Characteristic (ROC) curve for the VAD failure prediction model. The curve illustrates the model’s discriminative ability in the training dataset, with an area under the curve (AUC) of 0.723 (95% CI 0.637–0.810). The model maintained similar discrimination on the 30% hold-out validation set (AUC 0.764).

We established a scoring system based upon the above-mentioned multivariable logistic regression analysis to simplify the evaluation of the risk of failed VAD. Employing a positive threshold of ≥1.5, the risk score demonstrated a sensitivity of 61.5% and a specificity of 76.2%. A ROC curve (Figure 2) revealed that the model had predictive capabilities for a failed VAD, as indicated by an AUC of 0.723 (95% CI 0.637–0.810). The model demonstrated good calibration, with no evidence of lack of fit on the Hosmer–Lemeshow goodness-of-fit test (χ^2^ = 6.73, df = 8, *p* = 0.566).

Internal validation using a randomly selected subset comprising 30% of the cohort was conducted to assess the predictive performance of the score. The model demonstrated discriminative ability on this validation set, with an AUC of 0.764 (95% CI 0.619–0.909; *p* < 0.001).

## 4. Discussion

The safe management of the second stage of labor is critical, and unnecessary interventions should be avoided. However, the decision to perform a vacuum-assisted delivery is often based upon subjective assessment and clinical expertise, and deeming it unsuccessful and time to revert to a CD can often pose a challenge. The goal of our study was to identify risk factors for a failed VAD and develop a predictive model to aid in expeditious clinical decision-making.

### 4.1. Principal Findings

1. Induction of labor, a prolonged (>3.5 h) second stage of labor, fetal head station below +2 cm relative to the ischial spines, preeclampsia, newborn birthweight > 3750 gr, and newborn male gender were associated with increased risk for failed VAD, whereas maternal age > 35 years was associated with lower risk for VAD failure.
2. A prediction score model produced predictive performance with an AUC of 0.723 (95% CI 0.637–0.810).

There are several retrospective studies on VAD failure in medical literature. In a large Swedish study on 4747 failed VAD cases and 83,671 successful VAD procedures, Ahlberg et al. found that among other factors, medical induction of labor, male gender, and increased birth weight were risk factors for failed VAD [11]. Verhoeven et al. studied 6734 VAD trials, of which 309 had failed [14]. Their study showed that increased birthweight > 3750 g, augmentation of labor, and lower presenting part station compared to ischial spines were independent risk factors for a failed VAD.

Palatnik et al. compared 4352 women who had OVD by means of vacuum or forceps extraction that failed in 272 of them [15]. Although those authors did not distinguish between devices, they did identify a higher presenting part station as an independent risk factor for a failed OVD. Notably, Tutschek et al. observed that vaginal examination during labor may fail to accurately determine the fetal head station in nearly one-half of the cases [19].

Numerous studies have linked male gender to an increased risk of a failed OVD [16,20], particularly a failed VAD [11]. Melamed et al. investigated the effect of fetal gender on pregnancy outcomes in a study involving 66,387 deliveries. Their findings confirmed that male gender significantly increased the risk of a failed OVD [20]. Similarly, Sainz et al. observed a clear link between male gender and an increased risk of OVD failure [16]. Furthermore, Melamed et al. also reported significant differences in head measurement growth patterns between males and females, with males having larger head circumferences [21], supporting the findings of Kabiri et al. who suggested that a head circumference greater than the 90th percentile was significantly associated with an increased risk of failed VAD [22]. We did not evaluate head circumference, but our findings on fetal gender and weight support these studies.

Interestingly, preeclampsia emerged as an independent risk factor for failed VAD in our cohort. To the best of our knowledge, the association between preeclampsia and VAD failure has not been specifically evaluated in earlier reports. However, previous evidence suggested that preeclampsia may increase the likelihood of requiring a VAD in the setting of non-progressive labor [23], and a large population-based Swedish study similarly identified preeclampsia as a significant risk factor for VAD [24]. A plausible explanation is that preeclampsia is associated with placental dysfunction and reduced fetal reserve, which may increase the incidence of non-reassuring fetal heart rate patterns and the need for expedited delivery. In addition, preeclampsia has been linked to impaired uteroplacental perfusion and intrapartum fetal intolerance, potentially lowering the threshold for operative intervention and increasing the risk of an unsuccessful VAD.

While numerous studies have linked the occiput posterior (OP) position with an increased risk of a failed VAD [11,12], our findings, like those of Ashwall et al. [25], did not support such an association. One possible explanation lies in the difficulty of accurately assessing fetal head position by means of a clinical examination, particularly with regard to the OP position, which has been shown in numerous studies to be incorrect in up to 50% of the time when compared to ultrasound assessments [26,27]. Moreover, Akmal et al. found that although 20% of fetuses were in an OP position during transabdominal sonography carried out at full cervical dilation, fewer than one-half of them maintained that position until delivery [27]. This discrepancy could be attributed to the increasing use of intra-partum sonography to determine fetal head position, potentially resulting in more precise cup placement [26,27]. Intra-partum sonography might also improve selection of candidates for a VAD, potentially contributing to the comparatively lower rate of VAD failure in our cohort compared to other reports [5,6].

Several authors have created statistical modules of varying complexity to predict VAD success rates. For example, Verhoeven et al. developed a prediction model based upon a case–control study carried out in 2 medical centers in The Netherlands, revealing a failure rate of up to 5% [14]. However, their model did not incorporate other factors that emerged as significant predictors in our cohort, particularly the duration of the second stage of labor. Sainz et al. developed a model for a failed OVD based upon sonographic evaluations in a cohort of only 84 parturients [16]. Palatnik et al. presented several risk factors for a failed OVD but did not incorporate them into a predictive model [15].

### 4.2. Strengths and Limitations

This study has several important strengths. It is based on a large contemporary cohort of VAD attempts collected over a prolonged period in a high-volume tertiary center, enabling robust evaluation of a relatively uncommon outcome such as failed VADs. The use of a comprehensive and standardized institutional database allowed the inclusion of a wide range of maternal, obstetric, and intrapartum variables and reduced the risk of incomplete data capture. In addition, the proposed prediction score was derived from routinely available clinical parameters, which enhances its potential applicability in real-world obstetric practice. The model was internally validated using a split-sample approach and demonstrated stable discriminatory performance, supporting the robustness and consistency of the findings. Furthermore, the outcome of failed VAD was clearly and operationally defined, reducing misclassification bias and improving the clinical interpretability of the results.

Several limitations should also be considered when interpreting the findings. The retrospective design is inherently associated with potential selection bias, inaccuracies in recorded data, and limited control over exposures and outcomes. Detailed information on clinically relevant but difficult-to-measure factors, such as traction force, degree of fetal engagement, uterine contractility, cup placement technique, and operator experience, was not available in the database. These variables are subjective, lack standardized documentation, and are challenging to quantify reliably, which limits their incorporation into a clinically applicable prediction model.

The study was conducted in a single tertiary care center with a relatively homogeneous population and specific clinical protocols, which may limit the generalizability of the findings to other institutions, populations, and practice settings. In addition, several adverse neonatal outcomes were rare events, resulting in wide CIs; therefore, these findings should be interpreted with caution and primarily as descriptive rather than precise estimates of effect.

Intrapartum sonographic parameters were not included in the present analysis, although previous studies suggest that ultrasound assessment during labor may improve the evaluation of labor progression and prediction of delivery outcomes. The incorporation of such objective measures may further enhance predictive accuracy in future models.

Although internal validation demonstrated stable model performance, the model was developed and validated within the same retrospective cohort and may therefore be considered of value. External validation in independent cohorts and diverse clinical settings is required before clinical implementation to confirm reproducibility, calibration, and generalizability. Further research should also evaluate the clinical utility of the score across different decision thresholds and its potential impact on counseling, shared decision-making, and obstetric outcomes.

Finally, the model demonstrated moderate sensitivity, indicating that a proportion of failed VAD cases may not be identified by the score. Accordingly, the proposed model should be interpreted as a supportive risk stratification tool that complements clinical assessment rather than serving as a standalone determinant of clinical management.

## 5. Conclusions

We developed a model for predicting the likelihood of VAD failure that has the potential to assist obstetricians in clinical decision-making of whether to abandon VAD and proceed to a CD according to specific parturient and fetal characteristics. Future external validation and prospective evaluation are required to confirm generalizability and to assess the clinical utility of the score.

## Figures and Tables

**Table 1:** Maternal characteristics of study and control groups.

Characteristic	Failed Vacuum Delivery (n = 172)	Successful Vacuum Delivery (n = 8713)	*p*-Value
Maternal age at delivery (y), mean (±SD)	31.5 (4.3)	31.9 (4.6)	0.264
Maternal age >35 years, n (%)	28 (16.3)	2028 (23.3)	0.031
Nulliparity, n (%)	167 (97.1)	8068 (92.6)	0.025
Pregnancy via IVF, n (%)	32 (18.6)	796 (9.1)	<0.001
Pregestational BMI kg/m^2^, IQR (25–75%)	22.6 (20.2–25.6)	21.6 (19.8–24.0)	0.429
Pregestational BMI ≥ 30, n (%)	6 (3.5)	376 (4.3)	0.596
GWG (kg), IQR (25–75%)	13.5 (10.0–16.0)	13.0 (10.0–16.0)	0.305
Maternal height (cm), IQR (25–75%)	162 (158–168)	163 (160–158)	0.645
Pregestational diabetes, n (%)	0	40 (0.5)	0.373
Gestational diabetes, n (%)	19 (11.0)	796 (9.1)	0.39
Preeclampsia, (%)	7 (4.1%)	139 (1.6%)	0.011
Smoking, n (%)	6 (3.5)	449 (5.2)	0.327

IQR = interquartile range; IVF = in vitro fertilization; BMI = body mass index; GWG = gestational weight gain; CD = cesarean delivery.

**Table 2:** Obstetrical characteristics of study and control groups.

Characteristic	Failed Vacuum Delivery (n = 172)	Successful Vacuum Delivery (n = 8713)	*p*-Value
Epidural anesthesia, n (%)	138 (80.2)	8005 (91.9)	<0.001
Induction of labor, n (%)	62 (48.8)	2628 (30.6)	<0.001
Length of second stage of labor (hours) IQR (25–75%)	3.15 (2.12–4.09)	2.25 (1.21–3.1)	<0.001
Fetal head station below +2 cm relative to the ischial spines, n (%)	41 (64.1)	2354 (45.8)	0.004
PROM, n (%)	5 (2.9)	646 (7.4)	0.024
Meconium-stained amniotic fluid, n (%)	43 (25.0)	2231 (25.6)	0.857
Second stage > 3 h, n (%)	63 (36.6)	2633 (30.2)	0.07
Second stage > 3.5 h, n (%)	49 (28.5)	1234 (14.2)	<0.001
Occiput posterior, n (%)	27 (15.7)	861 (9.4)	0.009
Gestational age (wk), IQR (25–75%)	40.3 (39.4–41.1)	40.0 (39.1–40.5)	<0.001
Gestational age > 41 wks, n (%)	50 (29.1)	1789 (20.5)	0.006
Birthweight (gr), mean (±SD)	3375 (±398)	3240 (±410)	0.745
Birthweight > 3750 gr, n (%)	31 (18.0)	905 (10.4)	0.001
Male newborn, n (%)	119 (64.7)	5199 (56.6)	0.028

IQR = interquartile range; SD = standard deviation; AROM = artificial rupture of membranes.

**Table 3:** Neonatal Outcomes in the Study and Control groups.

Outcome	Failed Vacuum Delivery (n = 172)	Successful Vacuum Delivery (n = 8713)	*p*-Value
NICU, n (%)	35 (20.3)	500 (5.7)	<0.001
IVH, n (%)	0	5 (0.1)	0.753
Seizures, n (%)	3 (1.7)	113 (1.3)	0.609
Skull fractures, n (%)	1 (0.5)	3 (0)	<0.001
Subgaleal hematoma, n (%)	9 (5.2)	24 (0.3)	<0.001
Intracranial hemorrhage, n (%)	0	1 (0)	0.888
Neonatal death, n (%)	1 (0.6)	5 (0.1)	0.009

NICU = neonatal intensive care unit; IVH = intraventricular hemorrhage.

**Table 4:** Multivariate Analysis for Failed Vacuum Assisted Delivery.

Variable	β Coefficients	OR	95% CI	*p*-Value	Yes/No	
Medical induction of labor	0.625	1.87	1.02–3.43	0.044	0	0.5
Length of 2nd stage >3.5 hr	0.96	2.61	1.28–5.30	0.008	0	1
Fetal head station below +2 cm relative to the ischial spines	0.895	2.35	1.33–4.52	0.004	0	1
Newborn birthweight >3750 gr	0.802	2.23	1.06–4.70	0.035	0	1
Preeclampsia	2.018	7.52	2.77–20.42	<0.001	0	2
Male gender	0.675	1.96	1.02–3.78	0.043	0	1
Maternal age >35 years	−0.885	0.55	0.17–1.02	0.055	NA	
IVF mode of conception	0.482	1.62	0.60–4.38	0.342	NA	
Occiput posterior position	0.348	1.42	0.59–3.41	0.436	NA	
Epidural anesthesia	−0.415	1.42	0.59–3.41	0.401	NA	
PROM	−0.497	0.61	0.15–2.56	0.497	NA	
Gestational age >41 weeks	−0.065	0.94	0.46–1.91	0.859	NA	

IVF = in vitro fertilization; PROM = premature rupture of membranes.

## Data Availability

Data supporting this study’s findings are available from the corresponding author upon reasonable request.
